# Randomized double-blinded pilot clinical study of the antidiabetic activity of *Balanites aegyptiaca* and UPLC-ESI-MS/MS identification of its metabolites

**DOI:** 10.1080/13880209.2017.1354388

**Published:** 2017-07-20

**Authors:** Hend Rashad, Fateheya M. Metwally, Shahira M. Ezzat, Maha M. Salama, Adel Hasheesh, Amira Abdel Motaal

**Affiliations:** a Department of Environmental and Occupational Medicine, National Research Center, Dokki, Egypt;; b Department of Pharmacognosy, Faculty of Pharmacy, Cairo University, Giza, Egypt;; c Department of Research on children with special needs, National Research Center, Dokki, Egypt

**Keywords:** *Balanites aegyptiaca*, UPLC profiling, antidiabetic activity, clinical study

## Abstract

**Context:***Balanites aegyptiaca* Del. (Zygophyllaceae) fruits are traditionally known for the treatment of hyperglycaemia. Several *in vitro* and *in vivo* studies proposed some mechanisms of action. However, clinical trials in human beings were never reported to date.

**Objectives**: To investigate the antidiabetic efficacy of the 70% ethanol extract of the pericarps of *B. aegyptiaca* (BE) within a nutritional intervention in elderly people.

**Materials and methods:** Ultra-performance electrospray ionization-mass spectroscopy (UPLC-ESI-MS/MS) analysis was used for metabolic profiling of BE which was incorporated in hard gelatine capsules (400 mg/day) and tested on 30 type 2 diabetes (T2D) Egyptian patients for 8 weeks. According to sex, age and body mass index participants were divided into two equivalent groups, placebo and treatment.

**Results:** Thirteen compounds were identified in BE using UPLC-ESI-MS/MS analysis among which five steroidal saponins, seven phenolic compounds and a sterol glucoside. At the end of the 8-week treatment, the treated group showed 26.88% decrease in 2 h postprandial plasma glucose relative to 2.6% increase in the placebo group, while fasting plasma glucose was reduced to 10.3%. Treatment with BE capsules for 8 weeks produced significant reduction in the plasma triglyceride, total cholesterol and low-density lipoprotein cholesterol by 9.0, 12.76 and 21.35%, respectively, with 29.8% increase in the high-density lipoprotein cholesterol. Plasma alanine transaminase and aspartate transaminase were reduced by 42.6 and 43.3%, respectively.

**Discussion and conclusion:** Administration of the BE capsules to T2D resulted in significant improvements in the glycaemic markers and the lipid profile, without adverse effects or hypoglycaemia.

## Introduction

Diabetes mellitus (DM) is a growing public health problem in both developed and developing countries. The World Health Organization (WHO) reported that 422 million adults suffered from diabetes in 2014, compared to 108 million in 1980. About 3.7 million patients died from diabetes and diabetes-related complications in 2012 (WHO 2016). In general, two types of diabetes are recognized: type 1 diabetes (T1D) or insulin-dependent DM, and type 2 diabetes (T2D) or non-insulin-dependent DM. Complications like nephropathy, neuropathy, retinopathy, dyslipidaemia and cardiovascular diseases can be observed over time with T1D and T2D (Deshpande et al. 2008).

Oral hypoglycaemic drugs exert their action either by increasing the sensitivity to insulin as biguanides and thiazolidinediones or by increasing insulin secretion as sulphonylureas and meglitinides, others act as α-glucosidase inhibitors, incretin agonists and dipeptidyl peptidase-4 inhibitors (Lorenzati et al. 2010). Although oral hypoglycaemic drugs/insulin treatment can control the early complications of diabetes, serious late complications may emerge in patients (Tzoulaki et al. 2009). Some T2D patients suffer from fat accumulation in peripheral tissues, muscles, eyes and pancreas (Campbell-Tofte et al. 2007). This effect is due to interference with insulin secretion and signaling that can lead to ectopic lipid accumulation and disorders in metabolism (Roden 2005). Unfortunately, no cure could be found for T2D or for preventing its complications as most of the currently used oral antidiabetic drugs work through reducing fasting plasma glucose (FPG) and glycosylated haemoglobin (HbA1c) (Koski 2006).

Hypoglycaemia, lactic acidosis, peripheral oedema and abdominal discomfort usually accompany the use of the aforementioned oral hypoglycaemic drugs (Lorenzati et al. 2010). Hence, the search for new effective antidiabetic agents with fewer side effects is ongoing. Medicinal plants have always been an important source for finding new remedies for human health problems. Traditionally, numerous herbs were recommended for treatment of diabetes. Antidiabetic effects of many plants have been reported. However, these reports were confirmed by animal models and *in vitro* studies but limited evidence exists about their clinical usefulness.

*Balanites aegyptiaca* Del. (Zygophyllaceae), known as the desert date, is a spiny, evergreen tree commonly grown in the arid regions of Africa, the Middle East and Southern Asia (Hall 1991). In Egyptian folk medicine, the fruits possess an ethnopharmacological use as an oral hypoglycaemic (Kamel 1998). *Balanites* fruits revealed significant antidiabetic activities in STZ-induced diabetic rats and mice (Kamel et al. 1991; Gad et al. 2006). A previous study, by members of our group, showed that the reported antihyperglycaemic activity of *B. aegyptiaca* could be attributed to significant insulin-like and partly glitazone-like activities in cultured C2C12 skeletal muscle cells and 3T3-L1 adipocytes, respectively (Abdel Motaal et al. 2012). Later on, our group revealed that the saponin-rich fraction of *Balanites* and its content of furostanol saponin derivatives possessed significant *in vitro* aldose reductase inhibitory activities (Abdel Motaal et al. 2015).

In the present study, a randomized double-blinded pilot clinical trial was designed in order to evaluate the efficacy and safety of the Egyptian traditional plant *B. aegyptiaca* for the treatment of T2D in patients receiving oral antidiabetic medication. Sugar and lipid metabolic parameters in addition to serum markers of liver and kidney functions were monitored. Furthermore, ultra-performance electrospray ionization-mass spectroscopy (UPLC-ESI-MS/MS) profiling of the ethanol extract of *B. aegyptiaca* (BE) was performed.

## Material and methods

### Study design

This study was designed as a randomized, crossover, double-blind, placebo-controlled 8-week study and conducted at the Department of Nutrition at the National Research Center, Cairo, Egypt. It was reported according to the recommendation of the Consolidated Standards of Reporting Trials (CONSORT) (Schulz et al. 2010). The study protocol was reviewed and approved by the National Research Center (NRC) Ethical Committee (registration no. N-RC-20034456).

The participants received either 400 mg of BE (1 capsule per day) or placebo capsules (1 capsule per day) before lunch during the treatment period. The placebo capsules were composed of potato maltodextrin. An independent statistician prepared sequential sealed envelopes based on a random number table generated using SAS 9.2 (SAS Institute, Cary, NC). Randomization was implemented without blocks. The envelopes and the allocation sequence were managed by a statistician. The empty capsules employed were supplied by Arab Gelatin and Pharmaceutical Products, Egypt. Extract and placebo capsules were indistinguishably packaged and labelled with a subject number. Neither the research personnel, who treated or assessed the subjects, nor the subjects were aware of the subjects’ treatment assignments until the double-blind study was completed. The manuscript was in accordance with CONSORT checklist (supplementary file).

### Participants

**Figure 1.**
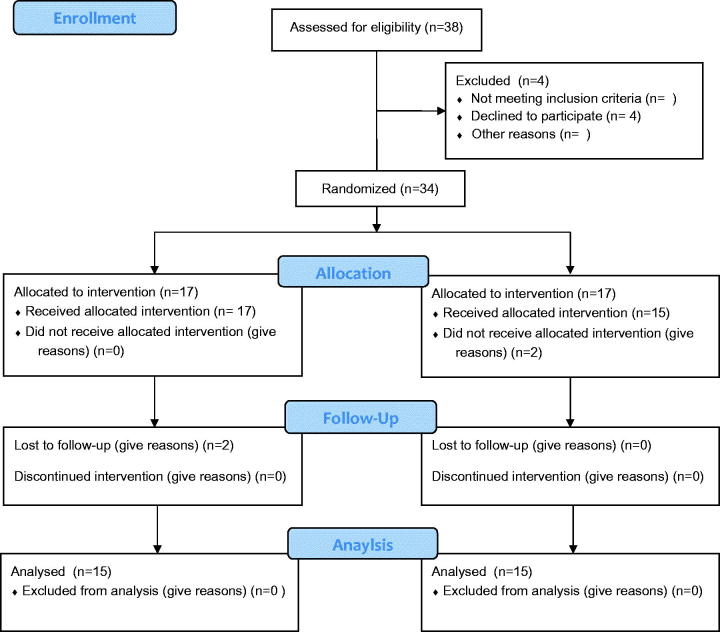
CONSORT 2010 flow diagram.

Thirty T2D patients (17 males and 13 females), aged 50–62 years, were recruited from 38 responders to an announcement at the NRC and Faculty of Pharmacy, Cairo University from January to March 2015. The average duration of the disease in the participants was 6.1 ± 2.2 years. Twenty-three participants were under oral antidiabetic drugs control: 10 used metformin, 13 received sulphonylureas and nine were taking antihypertensive drugs. Exclusion criteria were as follows: (1) T2D patients on insulin medication, (2) patients suffering from allergies and (3) those with recent thrombotic cases or having uncontrollable hypertension. Before study, participants received individual consultations from a clinical nutritionist to know the best way for controlling their daily calorie intake. They were asked to record their blood glucose and blood pressure as well as intake of other medications daily at home. Allocation of the participants was according to CONSORT flow diagram (Figure 1).

### Plant material

*B. aegyptiaca* fruits were brought from Aswan in February 2014 and authenticated by Dr. M. Gebali (Plant Taxonomy and Egyptian Flora Department, National Research Center, Giza, Egypt). A voucher specimen (voucher no. 201) was deposited at the herbarium of the Pharmacognosy Department, Faculty of Pharmacy, Cairo University, Egypt.

### Preparation of the extract and encapsulation

The *Balanites* fruits (10 kg) were macerated in 70% ethanol (3 × 6 L) till exhaustion. The solvent was evaporated under reduced pressure at 40 °C to yield 5 kg of the dried ethanol extract (BE). The extract was then formulated into hard gelatine capsules. The composition of the capsules is shown in Table 1.

**Table 1.** The composition of *B. aegyptiaca* hard gelatine capsules.

Active ingredients	mg/capsule	Function	Textbook/Monograph
*Balanites* extract (BE) standardized to contain not less than 8.63 ± 0.2 μg/ml of stigmaserol-3-*O*-β-D-glucoside. Part used: pericarps Extraction solvent: 70 % ethanol Ratio of herbal drug to extract: 2:1	400		In-house specification
Inactive ingredients			
Avicel PH 101 (microcrystalline cellulose)	30	Diluent	USP37 NF 32
Aerosil 200 (colloidal silicon dioxide)	75	Anticaking agent	USP37 NF 32
Magnesium stearate	5	USP37 NF 32	Lubricant
Average filling weight	510		

Form, hard gelatine capsule; average filling weight, 510 mg ±10 %.

### Standardization of BE extract

HPLC analysis of the hard gelatine capsules of BE was performed on an Agilent 1100 series HPLC system, equipped with an Agilent G1311A quaternary pump, a G1314A variable wavelength detector and a G1328A manual injector. The column was an Agilent RP C18 – 5 μm, 250 × 4.6 mm. The mobile phases were methanol (A) and water (B). The separation was performed using gradient elution from 40% to 100% A in B for 30 min and kept isocratic for 10 min. UV detection was at 213 nm, flow rate was at 1 mL/min, and the injection volume was 10 μL.

A standard calibration curve was established using five concentrations of stigmasterol-3-*O*-β-D-glucopyranoside (2, 4, 6, 8 and 10 μg/mL). The standard stigmasterol-3-*O*-β-D-glucopyranoside (14.0 mg) was dissolved in 100 mL of 20% methanol in chloroform. This solution (1.0 mL) was transferred into a volumetric flask and the volume was completed to 10 mL with acetonitrile. Serial dilutions were prepared from this stock solution.

### Sample preparation

The content of 10 BE capsules was emptied and mixed well, and then 100 mg was weighed, dissolved in 100 mL of 20% methanol in chloroform and sonicated for 30 min. The stock solution (1 mL) was transferred into a 25-mL volumetric flask and the volume was completed with acetonitrile. The solution was then filtered through a membrane filter PTFE 0.45 μM and used for analysis.

### UPLC-ESI-MS/MS analysis of BE extract

Chromatographic separation was performed on an Agilent 6420 triple-quad UPLC system (Agilent Technologies, Palo Alto, CA) equipped with Acquity BEH shield RP 18 column (1500 × 2.1 mm, particle size 1.7 μm, Waters, Milford, MA). The method of Farag et al. (2015) was applied in which the following elution binary gradient was applied: 0–1 min, isocratic 95% A (water/formic acid, 99.9/0.1 [v/v]), 5% B (acetonitrile/formic acid, 99.9/0.1 [v/v]); 1–16 min, linear from 5 to 95% B; 16–18 min, isocratic 95% B; and 18–20 min, isocratic 5% B. The flow rate was 150 μL/min and the injection volume was 3.1 μL (full loop injection). Eluted compounds were detected from *m/z* 50 to 1000 using a MS QqQ mass spectrometer equipped with an electrospray ion source in negative ion mode. The following instrument settings were used: nebulizer gas, nitrogen, 40 psi; dry gas, nitrogen, 10 mL/min, 300 °C; capillary, −3000 V (+4000 V); end plate offset, −500 V; funnel 1 RF, 200 Vpp; funnel 2 RF, 200 Vpp. Metabolites were characterized by their retention times relative to external standards, their mass spectra and in comparison with reference literature.

### Biochemical assessment

Blood samples were collected from the participants pre-treatment and at the end of the 8-week treatment period. Plasma and serum samples were prepared and stored at −80 °C till analysis. Blood glucose (fasting and post prandial) was determined using an enzymatic colorimetric kit (Vitro Scient, Cairo, Egypt). Total cholesterol, triglyceride, high-density lipoprotein cholesterol (HDL-C) and low-density lipoprotein cholesterol (LDL-C) were estimated using an enzymatic colorimetric method from Centronic kit (Wartenberg, Germany). In addition, a commercial kit purchased from Biomed Diagnostics (White City, OR) was used for the estimation of the liver markers: aspartate transaminase (AST) and alanine transaminase (ALT). Kidney functions (urea and creatinine) were determined using the Cobas® Integra 800 analytical system (Roche Professional Diagnostics, Rotkreuz, Switzerland). HbA1c was measured using HPLC. Serum insulin and C-peptide levels were measured using ELISA Kit (BioSource, Leuwen, Belgium).

### Statistical analysis

The data were expressed as mean ± standard deviation. Statistical differences in the data from the two treatment groups were tested using the non-parametric Mann–Whitney *U*-test (GraphPad Prism software, San Diego, CA). The Friedman test for repeated measures and the associated Dunns’ post-test for pairwise comparisons were used for determining the differences within the groups for the measured parameters. Values of *p* < 0.05 were considered significant.

## Results

### Standardization of BE extract

**Figure 2.**
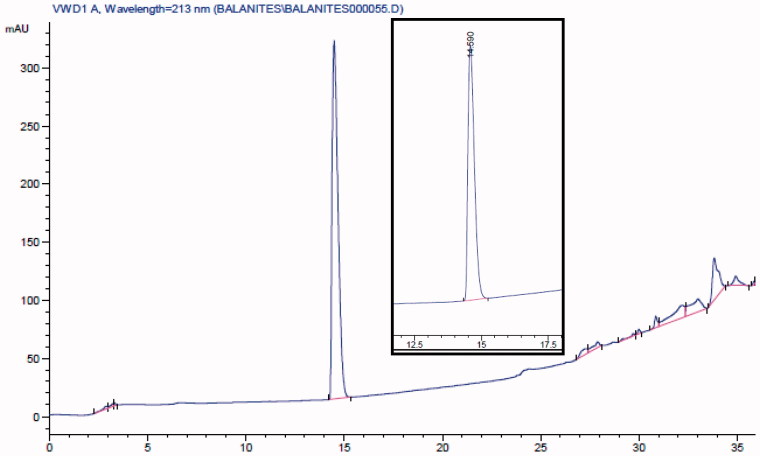
HPLC chromatogram of the 70% ethanol extract of *B. aegyptiaca* (BE) at 213 nm. The inset shows the standard stigmasterol-3 *O*-β-D-glucopyranoside at *R*_t_ = 14.6.min.

The BE capsules were standardized using RP-HPLC to contain not less than 8.63 ± 0.2 μg/mL of stigmaserol-3-*O*-β-D-glucoside (*R*_t_ = 14.6) (Figure 2).

### UPLC-ESI-MS/MS analysis of BE extract

**Figure 3.**
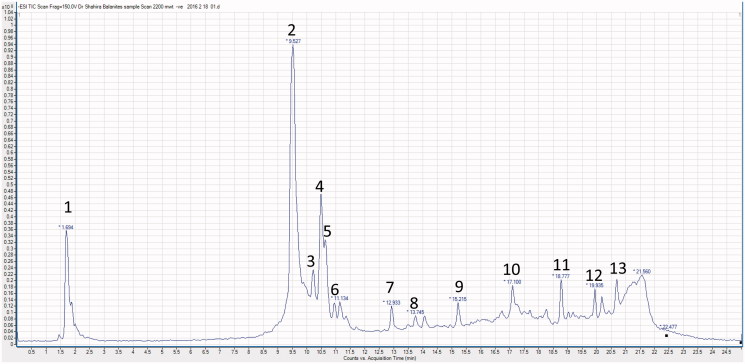
A representative UPLC-negative ionization MS trace of the 70% ethanol extract of *B. aegyptiaca* (BE).

The ESI-MS/MS spectra of the major peak (peak 2 at *R*_t_ 9.527 min) (Table 2 and Figure 3) has [M − H]^−^ at *m/z* 1063. The high molecular weight of this compound suggested that it is a saponin glycoside. The fragment ions at *m/z* 917 and 901 indicated the loss of 146 and 162 atomic mass unit (amu), corresponding to the loss of a deoxyhexose and a hexose, respectively. This loss also indicated that these are the two terminal sugars (Cui et al. 2000). The fragment ion at *m/z* 755 [M − H−hexose − deoxyhexose] corresponded to the loss of the two sugars. The fragment at *m/z* 431 showed the loss of two hexose units from the ion at *m/z* 755. Finally, a fragment ion at *m/z* 413 was correspondent with the loss of a H_2_O molecule. This fragment was consistent with a diosgenin nucleus (Kamel 1998). Comparing this data with the published literature, this compound was identified as the main saponin of *B. aegyptiaca* which is 26-*O*-glucopyranosyl-3,22,26-trihydroxyfurost-5-ene-3-*O*-glucopyranosyl-rhamnopyranosyl-glucopyranoside (Chapagain and Wiesman 2008).

**Table 2.** Peak assignments of metabolites in *B. aegyptiaca* extract (BE) using UPLC/ESI-MS/MS in negative ionization mode.

Peak No.	*R*_t_ (min)	[M−H]^−^	MS^2^	Metabolites
1	1.694	1209	1077, 1063, 915, 769, 589 and 431	26-*O*-glucopyranosyl-22-O-methylfurost-5-ene-3,26-diol-3-*O*-pentosyl-glucopyranosyl-rhamnopyranosyl-glucopyranoside
2	9.527	1063	917, 901, 755, 431 and 413	26-*O*-glucopyranosyl-3,22,26-trihydroxyfurost-5-ene-3-*O*-glucopyranosyl-rhamnopyranosyl-glucopyranoside
3	10.39	1195	917, 901, 977 and 413	26-*O*-glucopyranosyl-3,22,26-trihydroxyfurost-5-ene-3-*O*-glucopyranosyl-rhamnopyranosyl-glucopyranoside + xylose
4	10.50	1077	915, 769, 589 and 431	26-*O*-glucopyranosyl-22-*O*-methylfurost-5-ene-3,26-diol-3-*O*-glucopyranosyl-rhamnopyranosyl-glucopyranoside
5	10.75	1045	901, 883, 737, 593, 431 and 413	Diosgenin glycoside having two hexoses and two deoxyhexoses
6	11.134	465	303	Flavanonol-*O*-glucoside
7	12.93	609	301	Quercetin-3-*O*-rhamnoglucoside (rutin)
8	13.74	623	315	Isorhamnetin-*O*-rhamnoglucoside
9	15.21	477	315	Isorhamnetin-*O*-glucoside
10	17.10	301		Quercetin
11	18.77	574	412	Stigmasterol-3-*O*-glucoside
12	19.93	315		Isorhamnetin
13	20.62	341		Syringic acid

Peak 1 (*R*_t_ 1.694 min) showed the [M − H]^−^ at *m/z* 1209. The daughter ions at *m/z* 1077 and *m/z* 1063 were consistent with the loss of 132 and 146 amu, respectively. This indicated the loss of a pentose and a deoxyhexose which are the two sugars in the terminal residue of the sugar chain. The fragment ions from *m/z* 1077 to 431 indicated that this saponin peak was also a diosgenin glycoside, but has an additional pentose moiety and a methyl group. Upon comparing the obtained MS data with the published literature, this compound was closely related to 26-(*O*-glucopyranosyl)-22-*O*-methylfurost-5-ene-3,26-diol-3-*O*-pentosyl-glucopyranosyl-rhamnopyranosyl-glucopyranoside (Chapagain and Wiesman 2008).

Peaks 3, 4 and 5 at *R*_t_ 10.39, 10.5 and 10.75 min, respectively, showed [M − H]^−^ at *m/z* 1195, 1077 and 1045, respectively. The ESI-MS pattern of peak 3 (MS^2^ at *m/z* at 917, 901, 977 and 413) indicated a diosgenin nucleus. This compound is similar to the major one (peak 2) but has an additional pentose unit. For peak 4 [M − H]^−^ 1077, the MS^2^ ions showed the loss of 162; 308 (162 and 146 amu), 488 (two 162 unit, 146 and 18 amu) and 646 (three units of 162, 146 and 14 amu) from the parent ion (1077). The loss of 664 amu (three 162 units, 146, 14 and 18) from the parent ion gave rise to the ion at *m/z* 413 which is consistent with the dehydrated diosgenin (−H_2_O). This also suggested that the peak at *m/z* 1077 [M − H]^−^ is a methoxy derivative of the major compound (peak 2) and that the methoxy group is at position 22 as suggested before (Chapagain and Wiesman 2008). MS^2^ spectra of peak 5 with [M − H]^−^ at *m/z* 1045 showed the loss of 144 and 162 amu from the parent ion to give ions at *m/z* 901 and *m/z* 883, respectively. The fragment ion, at *m/z* 737, showed the loss of a hexose (162 amu) and a deoxyhexose (146 amu) from the parent ion. Another ion at *m/z* 593 indicated the loss of 144 amu which was previously reported in a furostanol saponin (Liang et al. 2002; Liu et al. 2004). This loss was corresponding to the cleavage of the E-ring. Further fragmentation of *m/z* 593 gave an ion at *m/z* 431 due to the loss of a hexose unit (162 amu). This compound was identified as a diosgenin glycoside having two hexoses and two deoxyhexoses (Chapagain and Wiesman 2008).

The peaks 6–9 were classified as flavonoid glycosides. Peak 6 appeared at *R*_t_ 11.134 min with [M − H]^−^ at *m/z* 465 and its MS^2^ showed a major peak at *m/z* 303 [M − H − 162]^−^. This indicated the loss of a hexose, most probably a glucose moiety. This compound was tentatively identified as flavanonol-*O*-glucoside (Zanutto et al. 2012). Peak 7 (*R*_t_ 12.93 min) with [M − H]^−^ at *m/z* 609, showed a major fragment ion at *m/z* 301 [M − H − 146 − 162]^−^ which indicated the loss of a rhamnose and a glucose moiety. The peak at *m/z* 301 corresponded to a quercetin aglycone. So, this compound was identified as quercetin-3-*O*-rhamnoglucoside (rutin). Both peak 8 (*R*_t_ 13.74 min) with [M − H]^−^ at *m/z* 623 and peak 9 (*R*_t_ 15.21 min) with [M − H]^−^ at *m/z* 477 had a main peak at *m/z* 315. This indicated an isorhamnetin aglycone after the loss of rahmnose and glucose moieties [M − H − 146 − 162]^−^ in case of peak 8 and the loss of a hexose moiety in case of peak 9 [M − H − 162]^−^. These compounds were identified as isorhamnetin-*O*-rhamnoglucoside and isorhamnetin-*O*-glucoside, respectively (Satterfield and Brodbelt 2001). The isorhamnetin aglycone (m/z 315) was also detected as peak 12 (*R*_t_ 19.93 min). Another flavonol aglycone was observed as peak 10 (*R*_t_ 17.10 min) with [M − H]^−^ at *m/z* 301 which was identified as quercetin. A phenolic acid was detected as peak 13 (*R*_t_ 20.62 min) with [M − H]^−^ at *m/z* 341 which was identified tentatively as syringic acid (Proestos et al. 2008). A steroidal glucoside was detected as peak 11 (*R*_t_ 18.77 min) with [M − H]^−^ at *m/z* 574. MS^2^ of the parent ion showed a major fragment ion at m/z 412 corresponding to a stigmasterol nucleus indicating the loss of a hexose [M − H − 162]^−^. This compound was identified as stigmasterol-3-*O*-glucoside.

### Randomized double-blinded clinical study

A total of 34 subjects were eligible and accordingly randomized to participate in the study. However, four subjects withdrew during the first period (two did not receive allocated intervention and two failed to follow-up). Accordingly, only 30 volunteers (17 men and 13 women) completed the 8-week double-blind trial. Recruitment started at the 1st of January and ended as planned by the 1st of March 2015. There were no statistical differences in baseline characteristics among the groups or between dietary parameters such as energy, protein, carbohydrate and fat intake (Table 3). Following the analysis of the pedometer readings, no differences in the physical activity were found between the groups. Consistent with previous findings, adverse effects were not observed as determined by physical examination, biochemical analysis and the comments received from the participants.

**Table 3.** Baseline parameters of the participants.

	Placebo group	BE-treated group
Baseline characteristics		
Males [*n* (%)]	8 (53.3 %)	9 (60.0 %)
Females [*n* (%)]	7 (46.7 %)	6 (40.0 %)
Age (years)	54.2 ± 5.2	54.9 ± 7.1
BMI (kg/m^2^)	28 ± 5	29.1 ± 3
Period since diagnosis	5.4 ± 3.2	6.8 ± 1.2
BP systolic (mm Hg)	128.8 ± 11.2	125 ± 13
BP diastolic (mm Hg)	79.8 ± 7.1	80.2 ± 10.5
HbA1c	6.7 ± 0.2	6.8 ± 2.1
No. of participants on medications		
Oral antidiabetic medication	12	11
Oral antihypertensive medication	5	4
Cholesterol reducing medication	3	2

Data are mean ± SD. There were no significant differences between the groups.

The baseline physical characteristics of the participants suggested that no adverse effects were detected in the BE-treated or placebo groups (Table 3). Moreover, no changes from baseline values were observed regarding kidney function markers, body weight and blood pressure in treated and placebo groups. After the 8-week treatment period, the BE-treated group showed a significant reduction in the postprandial plasma glucose (PPG) (2 h levels). This effect was more pronounced in the patients who started the study with HbA1c levels below 7.3%, i.e., having moderately elevated blood glucose levels. BE-treated group had a mean decrease in PPG by 26.9% versus only 2.6% decrease in placebo group (Table 4).

**Table 4.** Effect of 8-week treatment using BE capsules on the metabolic parameters.

	Placebo (*n* = 15)		Treated (*n* = 16)			
Parameter	Baseline	8 weeks	Baseline	8 weeks	*p*^a^	*p*^b^
Postprandial plasma glucose (mmol/l)	15.2 ± 4.1	15.6 ± 3.7	16.0 ± 2.4	11.7 ± 1.01	ns	0.003
Fasting plasma glucose (mmol/l)	8.2 ± 0.6	8.5 ± 1.1	8.7 ± 1.9	7.8 ± 0.9	0.05	0.04
Plasma – triglyceride (mg/dl)	181.7 ± 2.9	179.1 ± 8.5	172.4 ± 4.3	156.8 ± 5.1	0.05	0.003
Plasma – total cholesterol (mg/dl)	216.9 ± 11	217.1 ± 8.1	211.5 ± 6.8	184.5 ± 3.9	0.03	0.002
Plasma – HDL (mg/dl)	31.9 ± 8.6	32.8 ± 7.9	32.7 ± 2.9	46.6 ± 7.0	0.03	0.004
Plasma – LDL (mg/dl)	144.5 ± 5.2	140.1 ± 6.8	138.6 ± 11.1	109.0 ± 5.4	ns	0.003
LDL:HDL	4.5	4.27	4.2	2.33		
Serum insulin (mU/l)	11 ± 2.3	11 ± 4.6	10 ± 1.2	10 ± 1.7	ns	ns
Serum C-peptide (pmol/l)	476 ± 58.9	482 ± 63.1	493 ± 71.3	495 ± 69.2	ns	ns
Plasma – ALT (U/l)	39.1 ± 3.1	36.9 ± 4.6	36.4 ± 8.7	20.87 ± 8.7	0.05	0.004
Plasma – AST (U/l)	27.5 ± 9.0	28.1 ± 3.2	31.4 ± 10.1	17.8 ± 8.7	0.03	0.004
Plasma creatinine (μ mol/l)	0.71 ± 0.06	0.69 ± 0.01	0.88 ± 3.4	0.90 ± 8.7	0.01	ns
Plasma urea (mg/l)	18.3 ± 1.7	17.5 ± 1.8	15.5 ± 2.6	16.93 ± 8.7	0.01	ns

Data are mean ± standard deviation.

ns: not statistically significant.

*p*^a^: within-group statistical significance calculated for the data; *p*^b^: statistical significance from the placebo group.

Values of *p* < 0.05 were considered significant.

The FPG was also reduced in the BE-treated group (10.3% with respect to the baseline value), but there was no change in FPG in the placebo group. These occurred without affecting serum insulin and C-peptide levels (Table 4).

Furthermore, HDL levels were significantly increased in the BE-treated group by 29.8% relative to the placebo group. Furthermore, fasting plasma LDL was reduced in the BE-treated group by 21.4% relative to the baseline value. This reduction was significant when compared to the placebo group. There was also a significant reduction in the plasma triglyceride and total cholesterol in the BE-treated group by 9.0 and 12.7%, respectively, when compared to the baseline values. Regarding AST and ALT values, there was significant reduction in the BE-treated group by 42.6 and 43.3%, respectively, when compared to the placebo group (Table 4).

## Discussion

The present study was designed primarily to investigate the antidiabetic effect of BE on T2D elderly healthy subjects, focusing on 2 h plasma glucose level and FPG level as well as on adiposity-, liver- and kidney-related markers. The fruits of *B. aegyptiaca* are consumed traditionally in Egypt as a potent hypoglycaemic agent. Several *in vitro* and *in vivo* studies carried out by members of our group and other groups evidenced this hypoglycaemic effect (Kamel et al. 1991; Gad et al. 2006; Abdel Motaal et al. 2012, 2015). However, no controlled clinical trials were ever conducted to confirm these preliminary evidences. For this reason, a clinical study to demonstrate a long-term efficacy of the plant was essential. To the best of our knowledge, this study is pioneer in evaluating the effects of *B. aegyptiaca* within a nutritional intervention in elderly people (50–62 years).

In this pilot clinical study, the BE capsules (each containing 400 mg of the standardized BE, which is the calculated dose for humans) were daily administered to T2D patients for 2 months. Significant reductions in FPG and PPG were observed, without encountering any side effects as hypoglycaemia or changes in basal plasma insulin and C-peptide levels. According to the WHO, most of the participants were classified as overweight, with normal fasting insulin and C-peptide levels (WHO 2000).

The effect of BE capsules was more pronounced on PPG than on FPG. A remarkable improvement was observed in the glycaemic condition of the participants having slight to moderate elevated blood glucose (with HbA1c levels <7.3%). So PPG increment is responsible for 70% of the resulting hyperglycaemia in those patients (Monnier et al. 2003). On the other hand, moderate improvements in PPG were observed in the study subjects who suffered from poor controlled diabetes (HbA1c 7.3–9.3%). Therefore, longer treatment periods or higher doses will be required for patients with HbA1c > 7.3%.

The decrease in HDL-C level, in patients with T2D, could be attributed to increased catabolism of HDL lipoproteins (Duvillard et al. 2000). Furthermore, the cardiovascular protective role of HDL is thought to be mainly due to its role in reverse cholesterol transport (Sviridov and Nestel 2002). It is noteworthy to mention that HDL-C was significantly increased in BE-treated patients as compared to the placebo group. The recovery in HDL levels (and LDL reduction to near optimum levels) in the BE-treated group was more observed in three of the individuals who started the study with lower HDL levels. The overall effect was a significant improvement in the LDL:HDL ratio, as well as in the serum triglycerides and total cholesterol levels under the BE capsules treatment when compared to the placebo group.

The levels of AST and ALT were significantly reduced in the BE-treated group when compared to the baseline values. This effect was mainly detected in five of the participants who had elevated levels of AST and ALT. However, BE had no significant effect on urea and creatinine levels.

A 2-month administration of standardized BE capsules to Egyptian T2D patients led to significant improvements in both FPG and PPG without any side effects or weight loss. The treatment decreased LDL-C, triglycerides and cholesterol, and increased the HDL-C levels. Hence, the BE capsules induced a glycaemic control that could be attributable to its hypoglycaemic as well as hypolipidaemic effects.

Characterization of the BE extract was performed using UPLC-ESI-MS/MS technique with the aim of identifying the major metabolites in BE. Thirteen compounds were identified in BE by their molecular weight, mass fragmentation and through comparison with the published literature. Included were four furostanol saponins and a diosgenin glycoside, four flavonoid glycosides, two flavonol aglycones, a phenolic acid and a steroidal glycoside (Table 2). A biologically guided fractionation of *Balanites* fruits proved that the dichloromethane and ethyl acetate extracts standardized to contain rutin and isorhamnetin increased the basal glucose uptake in C2C12 skeletal muscle cells and accelerated triglyceride accumulation in 3T3-L1 pre-adipocytes (Abdel Motaal et al. 2012). Another study showed that five new furostanol saponins isolated from *Balanites* fruits exhibited pronounced aldose reductase inhibitory activities compared to quercetin (Abdel Motaal et al. 2015). These findings raise the possibility that the metabolites identified in BE (Table 2) might have contributed through different potential mechanisms of action to the clinical hypoglycaemic and hypolipidaemic effects exerted by the BE capsules in this study.

However, the present study has its limitations. Further analyses of key enzymes in the cellular metabolism are a prerequisite for a better understanding of how the metabolic processes were affected by the BE treatment. Also studies on a larger sample size might be necessary to adequately demonstrate the effects of BE on T2D. The potential benefits in herbal treatments can only be properly realized if the remedies were tested in randomized, controlled and blinded clinical trials (Gertsch 2009).

## Conclusion

This is a clinical support for the previous *in vitro* and *in vivo* claimed effects, suggesting that *B. aegyptiaca* may induce an improvement in insulin sensitivity through reduction in fats levels. Herein, we propose BE as a new natural remedy for treatment as well as improving the quality of life in T2D patients.

## Disclosure statement

We confirm that there are no known conflicts of interest associated with this publication and there was no significant financial support for this work that could have influenced its outcome.

## Supplementary Material

Amira_Abdel_Motaal_et_al_supplemental_content.zip
